# An *In Vitro* System Comprising Immortalized Hypothalamic Neuronal Cells (GT1–7 Cells) for Evaluation of the Neuroendocrine Effects of Essential Oils

**DOI:** 10.1155/2015/343942

**Published:** 2015-10-21

**Authors:** Dai Mizuno, Keiko Konoha-Mizuno, Miwako Mori, Kentaro Yamazaki, Toshihiro Haneda, Hironari Koyama, Masahiro Kawahara

**Affiliations:** ^1^Department of Forensic Medicine, Faculty of Medicine, Yamagata University, 2-2-2 Iida-Nishi, Yamagata-shi, Yamagata 990-9585, Japan; ^2^Research Institute of Pharmaceutical Sciences, Faculty of Pharmacy, Musashino University, 1-1-20 Shin-machi, Nishitokyo-shi, Tokyo 202-8585, Japan; ^3^School of Pharmaceutical Sciences, Kyushu University of Health and Welfare, 1714-1 Yoshino-cho, Nobeoka-shi, Miyazaki 882-8508, Japan

## Abstract

Aromatherapy and plant-based essential oils are widely used as complementary and alternative therapies for symptoms including anxiety. Furthermore, it was reportedly effective for the care of several diseases such as Alzheimer's disease and depressive illness. To investigate the pharmacological effects of essential oils, we developed an *in vitro* assay system using immortalized hypothalamic neuronal cells (GT1–7 cells). In this study, we evaluated the effects of essential oils on neuronal death induced by hydrogen peroxide (H_2_O_2_), aluminum, zinc, or the antagonist of estrogen receptor (tamoxifen). Among tests of various essential oils, we found that H_2_O_2_-induced neuronal death was attenuated by the essential oils of damask rose, eucalyptus, fennel, geranium, ginger, kabosu, mandarin, myrrh, and neroli. Damask rose oil had protective effects against aluminum-induced neurotoxicity, while geranium and rosemary oil showed protective activity against zinc-induced neurotoxicity. In contrast, geranium oil and ginger oil enhanced the neurotoxicity of tamoxifen. Our *in vitro* assay system could be useful for the neuropharmacological and endocrine pharmacological studies of essential oils.

## 1. Introduction

Trace nutrients contained in plants such as vitamins and minerals are essential for human health. Essential oils used in aromatherapy are also useful for health maintenance as with these trace nutrients contained in plants. Aromatherapy is one of the folk remedies that encompasses the use of essential oils derived from various types of plant sources for a variety of application methods and is widely used as a complementary and alternative therapy for symptoms including anxiety and depression [1]. Ohno-Sadachi et al. reported that the odor of lavender ameliorates the influence of stress on the brain mechanisms subserving the circadian rhythm of autonomic nervous activities and maintains their activity pattern during sleep [2]. Furthermore, bergamot may be useful as an aromatherapeutic agent for minimizing the symptoms of stress-induced anxiety, mild mood disorders, and cancer-related pain [3]. The effects of aromatherapy are theorized to result from the binding of chemical components in the essential oil to receptors in the olfactory bulb, impacting the brain's emotional center, the limbic system. The stimulated brain may affect physiological functions of the autonomic nervous, endocrine, and immune systems via hormones, neurotransmitters, and cytokines. Fragrance compounds and essential oils with sedative effects were found to influence the motility of mice in inhalation studies under standardized conditions [4]. The researchers also found significant levels of the fragrance compounds in the plasma after inhalation, suggesting that the effects of aromatherapy result from a direct pharmacological interaction rather than an indirect central nervous system relay.

The prevalence of senile dementia, such as Alzheimer's disease (AD) and vascular-type senile dementia (VD), is increasing worldwide. In senile dementia, marked death of neurons in the cerebral cortex and hippocampus is observed. However, the prime cause of neuronal death is identical. VD is a cerebrovascular disease and mainly occurs after transient global ischemia. It is widely accepted that excess zinc (Zn) which is secreted during ischemia conditions causes neuronal death and plays a central role in the pathogenesis of VD. Meanwhile, it is widely accepted that the oligomerization of *β*-amyloid protein (A*β*P) and its neurotoxicity are crucial for the pathogenesis of AD. We have demonstrated that aluminum enhanced the oligomerization of A*β*P. Epidemiological studies have suggested that aluminum (Al) in drinking water is a risk factor for AD. In recent years, the potential of nonpharmacological therapies such as aromatherapy for the prevention of senile dementia has been studied [5]. The efficacy of aromatherapy has been investigated in clinical trials and in physiological psychology studies and as the animal use alternative. In these studies, a variety of factors (e.g., the dose and the injection route of the essential oil and the preference and the mental condition of the human subjects) affect the experimental results, materially. Hence, to elucidate the pharmacological effects of aromatic oils, the development of an easily controlled evaluation system is needed.

Following this need, we attempted to evaluate the pharmacological effects of aromatic oils employing our previously developed rapid, sensitive, and convenient assay system for high-throughput screening of such substances using GT1–7 cells (immortalized hypothalamic neurons) [6]. The GT1–7 cells, which were developed by genetically targeting the tumorigenesis of mouse hypothalamic neurons, possess a number of neuronal characteristics, such as the extension of neurites, secretion of gonadotropin-releasing hormone (GnRH), and expression of receptors and neuron-specific proteins including the *β*-estradiol (E2) receptor (both the ER*α* and ER*β* subtype), microtubule associated protein 2 (MAP2), tau protein, neurofilament, synaptophysin, GABA_A_ receptors, dopamine receptors, and L-type Ca^2+^ channels [7]. These properties make the GT1–7 cell line an excellent model system for the investigation of neurotoxicity and endocrine disruption [8, 9]. The olfactory system also acts as a gateway to the external environment. Kanayama et al. have shown that intranasally administered metal ions were transported from the nasal cavity to the olfactory bulb via the olfactory nerve pathway and from there to other brain regions such as the hypothalamus and the hippocampus [10]. In the present study, we investigated the effects of essential oils on GT1–7 cytotoxicity induced by H_2_O_2_, Al, Zn, or the estrogen receptor (ER) antagonist (tamoxifen: TMX).

## 2. Materials and Methods

### 2.1. Reagents

TMX was purchased from Sigma Aldrich (St. Louis, MO, USA). This compound was diluted in dimethyl sulfoxide (DMSO) prior to use. H_2_O_2_ was purchased from Wako Pure Chemical Industries, Ltd. (Osaka, Japan). AlCl_3_, ZnCl_2_, and maltol were purchased from Tokyo Chemical Industry Co., Ltd. (Tokyo, Japan). Al was reacted with maltol to yield the Al-maltol complex to increase the cell membrane permeability of Al. AlCl_3_ and maltol were diluted in distilled water at a molar ratio of 1 : 3 and used as an Al-maltol complex solution. In this study, the concentration of Al equals the concentration of Al^3+^ in the Al-maltol complex solution. The essential oils were purchased from GAIA/NP (Tokyo, Japan) and diluted by adding 99.5% ethanol just before use. All of the essential oils used in this study are shown in Table 1.

### 2.2. Cell Culture

GT1–7 cells (provided by Dr. R. Weiner, University of California at San Francisco) were grown in Dulbecco's Modified Eagle's Medium/Nutrient Mixture F-12 Ham (DMEM/F-12) supplemented with 10% fetal bovine serum. After enzymatic digestion using trypsin, the cells were resuspended in a serum-free medium and plated onto culture plates [11]. The cells were cultured in a humidified incubator at 37°C and 7% CO_2_.

### 2.3. Cell Viability Assay

Cell viability was assessed as described previously [12]. Briefly, dissociated GT1–7 cells were plated onto 96-well culture plates at a concentration of 5 × 10^4^ cells per well in 200 *μ*L culture medium. Following a 24 h incubation, the cells were treated with essential oil at a final concentration of 25 ppm, and H_2_O_2_, ZnCl_2_, Al–maltol, or TMX was added immediately to the medium. The final concentrations of these compounds are shown in Figures 2–4. After 24 h exposure, cell viability was quantified using the WST-1 assay with the Cell Counting Kit (Dojindo, Kumamoto, Japan). The absorbance of the treated samples was measured against a blank control by using a microplate reader (iMark Microplate Absorbance Reader; Bio-Rad Laboratories, CA, USA) set at 450 nm and 620 nm detection and reference wavelengths, respectively.

### 2.4. Statistical Analyses

All statistical evaluations were performed using two-tailed Student's *t*-test with the KaleidaGraph software (Synergy software, PA, USA). A probability level (*P*) of <0.01 or <0.05 was considered significant.

## 3. Results and Discussion

### 3.1. The Effect of Essential Oils on the Viability of GT1–7 Cells

**Figure 1:**
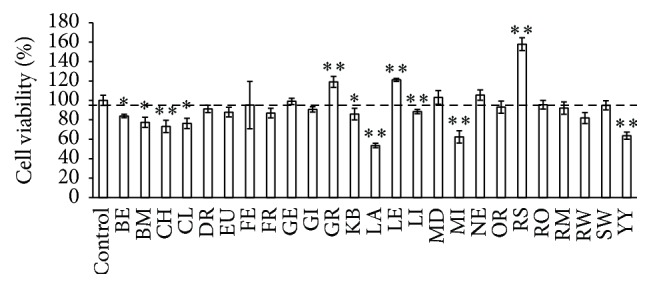
GT1–7 cell viability after exposure to various essential oils. GT1–7 cells were treated with 25 ppm of various essential oils. After 24 h, the viability was analyzed using the WST-1 method. Data are presented as the mean ± SEM (*n* = 6). ^*∗*^ *P* < 0.05, ^*∗∗*^ *P* < 0.01 versus the control group. The abbreviated names of the essential oils used in the figure are shown in Table 1.

We examined the effects of essential oils on the viability of immortalized hypothalamic neurons (GT1–7 cells).  Figure 1 shows the viability of cultured GT1–7 cells after a 24 h exposure to various essential oils. Several of the essential oils used in this study affect the viability of GT1–7 cells. We observed a significant increase in the viability of GT1–7 cells exposed grapefruit, lemon, or rosalina oil. On the other hand, the exposure to lavender, myrrh, or ylang ylang significantly decreased cell viability. These findings suggest that several essential oils may affect neuronal cells by a direct pharmacological interaction.

### 3.2. The Protective Activities of Essential Oils against Various Cytotoxic Agents

**Figure 2:**
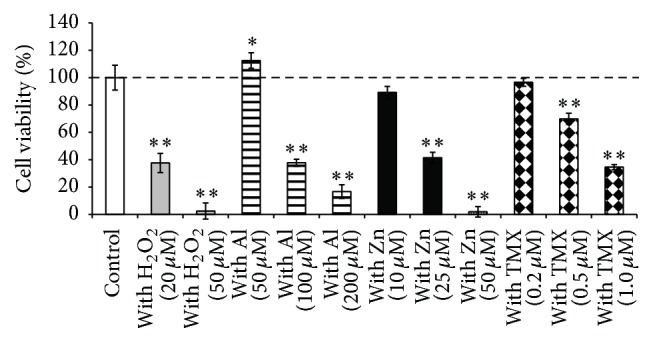
Cytotoxicity of H_2_O_2_, Al, Zn, and TMX. GT1–7 cells were treated with 20–50 *μ*M H_2_O_2_ (gray), 50–200 *μ*M Al (stripes), 10–50 *μ*M Zn (black), or 0.1–0.5 *μ*M TMX (lattice). After 24 h, the viability was analyzed using the WST-1 method. The white bar shows the viability of GT1–7 cells treated with essential oil alone. Data are presented as the mean ± SEM (*n* = 6). ^*∗*^ *P* < 0.05, ^*∗∗*^ *P* < 0.01 versus the control group. The abbreviated names of the essential oils used in the figure are shown in Table 1.

**Figure 3:**
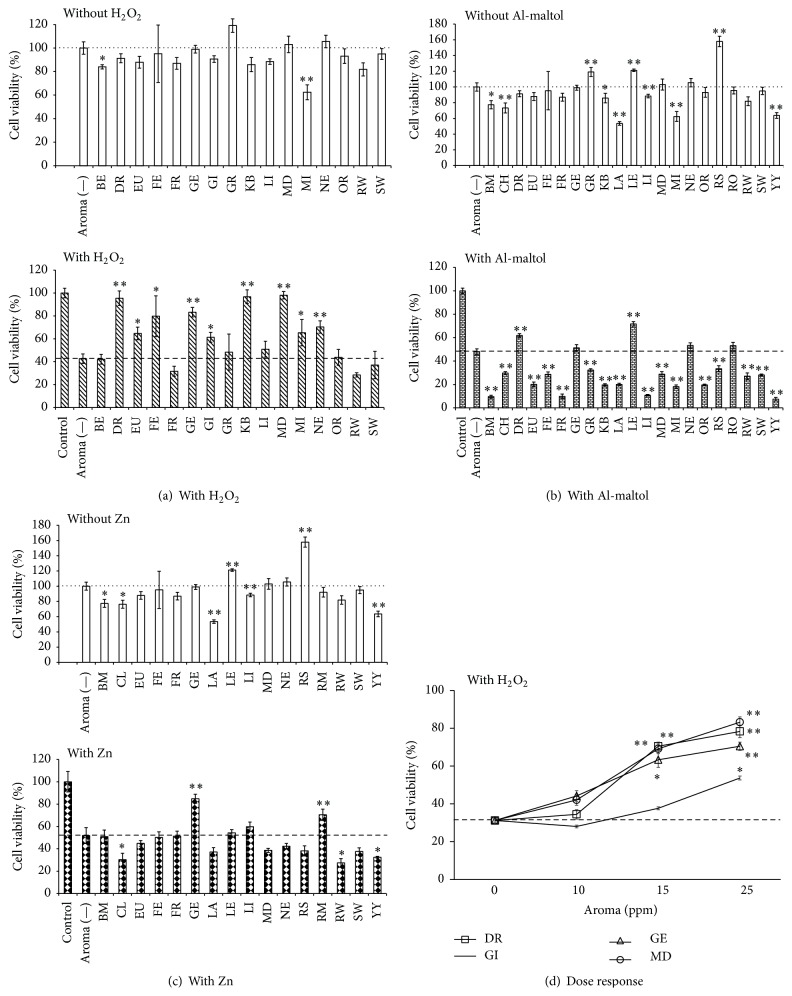
The effect of essential oils on GT1–7 cells exposed with various cytotoxins. GT1–7 cells were treated with 20 *μ*M H_2_O_2_ (a), 100 *μ*M Al (b), or 25 *μ*M Zn (c) without (aroma (—) in graph) or with 25 ppm of various essential oils. After 24 h, the viability was analyzed using the WST-1 method. The white bar shows the viability of GT1–7 cells treated with essential oil alone. Data are presented as the mean ± SEM (*n* = 6). (d) The dose dependency of the effects of essential oils on GT1–7 cell cytotoxicity. 10–25 ppm of essential oils was treated with 20 *μ*M H_2_O_2_. After 24 h, the viability was analyzed using the WST-1 method. Data are presented as the mean ± SEM (*n* = 3). ^*∗*^ *P* < 0.05, ^*∗∗*^ *P* < 0.01 versus the aroma (—) group. The abbreviated names of the essential oils used in the figure are shown in Table 1.

GT1–7 cells were treated with various essential oils and cytotoxic agents (H_2_O_2_, Al, and Zn) to investigate the protective activities of essential oils against neurotoxicity. H_2_O_2_ is known to produce a hydroxyl radical, thereby inducing cytotoxicity. Neuronal Al accumulation can result in neuron dysfunction and toxicity [13]. Furthermore, we previously reported that Zn is neurotoxic, causes neurodegeneration following transient global ischemia, and plays a crucial role in the pathogenesis of VD [10]. In this study, the abovementioned compounds caused cytotoxicity in a dose-dependent manner (Figure 2). Treatment with 20 *μ*M H_2_O_2_, 100 *μ*M Al, and 25 *μ*M Zn decreased GT1–7 cell viability to 42.2 ± 4.3%, 48.0 ± 2.5%, and 51.9 ± 7.0%, respectively (Figures 3(a)–3(c)).

As shown in Figure 3(a), damask rose, eucalyptus, fennel, geranium, ginger, kabosu, mandarin, myrrh, and neroli oil significantly increased the viability of H_2_O_2_-treated GT1–7 cells. However, these essential oils did not increase GT1–7 viability by themselves. These compounds may protect against H_2_O_2_-induced neurotoxicity. The protective effects of essential oils against H_2_O_2_-induced neurotoxicity increased in a dose-dependent manner.  Figure 3(d) showed some examples of these effects. Essential oil extracted from aromatic plant material is a mixture of various organic compounds. Numerous components of essential oils may be involved in these protective effects. For example, many of these oils contain terpene (e.g., geraniol, limonene, linalool, and *α*-pinene) (Table 1). It has been reported that many of these terpenes have antioxidative activity [14], which may contribute to the neuroprotective activities of these oils.

Damask rose and lemon oil increased the viability of Al-treated GT1–7 cells (Figure 3(b)). Damask rose oil protected against Al-induced neurotoxicity but had no effect on the viability of untreated cells. On the other hand, various essential oils, especially eucalyptus, frankincense, grapefruit, and orange oil, augmented Al-induced neurotoxicity. Some ingredients in these essential oils may be involved in the enhancement of Al-induced neurotoxicity or in intracellular Al accumulation. Exposure of Zn-treated GT1–7 cells to geranium or rosemary oil enhanced their viability, while exposure to clove, rosewood, or ylang ylang oil decreased their viability (Figure 3(c)). Geranium and rosemary oil may have a protective effect against Zn-induced neurotoxicity, because both of these oils did not affect general GT1–7 viability. As in the case of cotreatment with H_2_O_2_, the protective effects of essential oils against Al- or Zn-induced neurotoxicity increased in a dose- dependent manner (data not shown). Kawahara et al. have demonstrated that brain-derived neurotrophic factor (BDNF) markedly attenuated the Al-induced neurotoxicity, and Al inhibited the BDNF-induced elevation of intracellular calcium levels [15]. We have shown that ER stress and the disruption of calcium homeostasis are the underlying mechanisms of Zn-induced neurotoxicity [9]. Several histidine-related compounds, such as carnosine and anserine, protected against Zn-induced neurotoxicity by participating in the regulation of the ER-stress pathway and the activity-regulated cytoskeletal protein (Arc) [12, 16]. The coexistence of a membrane impermeable chelator such as Ca–EDTA with these metal ions can protect neurons from these metals that induced neurotoxicity by binding to metals in the culture medium and thereby inhibiting metal influx into the cells [12]. The investigation of the mechanisms underlying the neuroprotective activities of essential oils may lead to the development of therapeutic agents for several neurodegenerative diseases such as AD and VD.

### 3.3. The Effects of Essential Oils on Estrogen Receptor Mediated Cell Signaling

**Figure 4:**
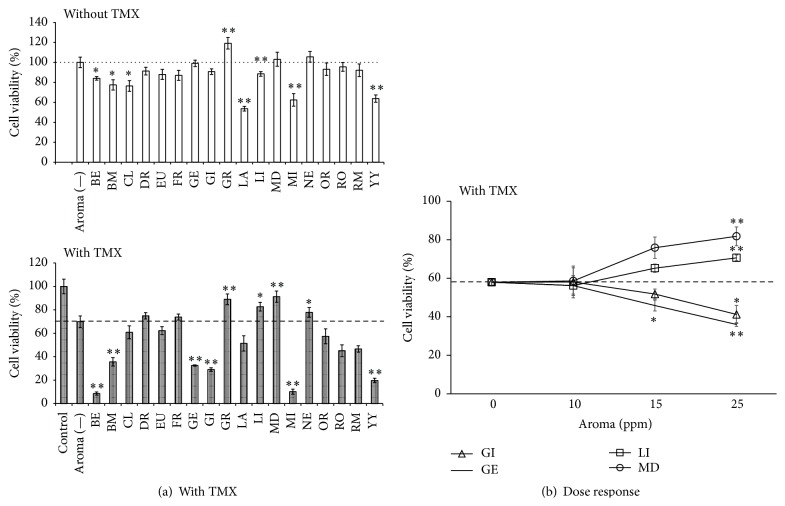
The effect of essential oils on TMX-induced GT1–7 cell cytotoxicity. (a) GT1–7 cells were treated with 0.5 *μ*M TMX without (aroma (—) in graph) or with 25 ppm of various essential oils. After 24 h, the viability was analyzed using the WST-1 method. The white bar shows the viability of GT1–7 cells treated with essential oil alone. Data are presented as the mean ± SEM (*n* = 6). (b) The dose dependency of the effects of essential oils on TMX-induced GT1–7 cell cytotoxicity. 10–25 ppm of essential oils was treated with 0.5 *μ*M TMX. After 24 h, the viability was analyzed using the WST-1 method. Data are presented as the mean ± SEM (*n* = 3). ^*∗*^ *P* < 0.05, ^*∗∗*^ *P* < 0.01 versus the aroma (—) group.

The GT1–7 cell line is also a useful model for the investigation of endocrine-disrupting effects, and the antagonist of ER (TMX) was used to induce GT1–7 cell death [17]. In this study, we evaluated caused cytotoxicity in a dose-dependent manner. Treatment with 0.5 *μ*M of TMX significantly decreased GT1–7 cell viability to 69.9 ± 4.6% (*P* < 0.01 versus the control group). The administration of lime, mandarin, or neroli oil significantly inhibited TMX-induced cytotoxicity. In contrast, administration of geranium or ginger augmented TMX-induced cytotoxicity without directly reducing GT1–7 cell viability (Figure 4(a)). The effects of essential oils on TMX-induced cytotoxicity increased in a dose-dependent manner.  Figure 4(b) showed some examples of these effects. The mechanisms underlying these effects of essential oils are under investigation. These essential oils may act as an agonist or an ER antagonist, and this hypothesis may be involved in the mechanism of the regulation of TMX-induced cell death by these essential oils. It has also been suggested that geranium oil affects ER-mediated signal regulation. Nakamura et al. reported that geranium oil might be useful for the amelioration of symptoms linked to premenstrual syndrome (PMS) [18]. E2 is involved in the regulation of the GnRH secretion through the conjunction with ER. Geranium oil may affect GnRH secretion through the ER, contributing to the moderating effect of this oil on PMS. Geranium oil also contains geraniol, which has been suggested to have estrogenic activity [19].

## 4. Conclusion

We showed the effects of various essential oils on multiple kinds of cytotoxicity using a convenient* in vitro* assay system. Essential oils contain various organic compounds such as terpenoid (see “major component” in Table 1). For example, limonene has been reported to have numerous beneficial effects, including antioxidant [20, 21] activities and promotion of neural cell differentiation [22]. Limonene is a monoterpene present in citrus essential oils and in many other essential oils (e.g., fennel, geranium, kabosu, myrrh, and neroli) and was shown to attenuate neuronal death induced by H_2_O_2_. Citronellol has been reported to have its antioxidant properties [23] and is present in damask rose, geranium, and rose. Various organic compounds in essential oils including these components may be involved in the pharmacological and protective effects of essential oils.

The GT1–7 cell line is a useful model of the neuroendocrine system, and the* in vitro* evaluation system using this cell line is a straightforward analysis system for the investigation of neurotoxicity and endocrine disruptive effects. In this study, several essential oils such as geranium oil showed neuroprotective and ER-mediating activities. Essential oil is a mixture of various organic compounds, and the effects of essential oils may be owing to the synergistic or competitive action of these compounds. The essential compounds and the underlying mechanisms of the effects of essential oils shown in this study need to be identified in the future. In conclusion, we hope that our screening method may be beneficial for the development of drugs for the treatment of neurodegenerative diseases or harmful endocrine disruptors.

## Figures and Tables

**Table 1:** Characteristics of the essential oils.

Name	Abbreviation in Figures	Scientific name	Major component
Benzoin	BE	*Styrax benzoin*	Benzoic acid, benzyl benzoate, benzyl alcohol
Bergamot	BM	*Citrus bergamia*	Linalyl acetate, limonene, linalool
Chamomile	CH	*Matricaria chamomilla*	Chamazulene, *α*-bisabolol, farnesene
Clove	CL	*Syzygium aromaticum*	Eugenol, *β*-caryophyllene, nerol
Damask rose	DR	*Rosa damascena *	Citronellol, geraniol, nerol
Eucalyptus	EU	*Eucalyptus globulus*	1,8-Cineole
Fennel	FE	*Foeniculum vulgare*	Anethole, fenchone, limonene
Frankincense	FR	*Boswellia carteri*	*α*-Pinene, dipentene, cadinene
Geranium	GE	*Pelargonium graveolens*	Citronellol, geraniol, limonene, *β*-caryophyllene
Ginger	GI	*Zingiber officinale*	*β*-Phellandrene, gingerol, *α*-zingiberene, *β*-zingiberene
Grapefruit	GR	*Citrus paradisi*	Limonene, myrcene, *α*-pinene
Kabosu	KB	*Citrus sphaerocarpa*	Limonene, myrcene
Lavender	LA	*Lavandula angustifolia*	Linalool, linalyl acetate
Lemon	LE	*Citrus limon*	Limonene, *α*-pinene, *β*-pinene
Lime	LI	*Citrus aurantifolia*	*p-*Cymene, *α*-terpineol, terpinolene
Mandarin	MD	*Citrus reticulata var. mandarin*	*γ*-Terpinene, *α*-pinene, *β*-pinene
Myrrh	MI	*Commiphora myrrha*	Cuminaldehyde, limonene, *α*-pinene
Neroli	NE	*Citrus aurantium var. amara*	Linalool, limonene, trans-ocimene
Orange	OR	*Citrus sinensis*	*d-*Limonene, myrcene, octanal
Rosalina	RS	*Melaleuca ericifolia*	Linalool, 1,8 cineol, *d-*limonene
Rose	RO	*Rosa*	Citronellol, geraniol
Rosemary	RM	*Rosmarinus officinalis *	Camphene, *α*-pinene, *β*-pinene
Rosewood	RW	*Aniba rosaeodora*	Linalool, *α*-terpineol
St. John's wort	SW	*Hypericum perforatum*	*α*-Pinene, *β*-pinene, germacrene D
Ylang Ylang	YY	*Cananga Odorata var. genuina *	Germacrene D, *β*-caryophyllene, methyl benzoate, linalool

## References

[1] Cooke B., Ernst E. (2000). Aromatherapy: a systematic review. *British Journal of General Practice*.

[2] Ohno-Sadachi H., Saitoh J., Nagai M. (2011). The odor of lavender maintains the pattern of autonomic nervous activities during sleep in humans exposed to stress. *Mt. Fuji Research*.

[3] Bagetta G., Morrone L. A., Rombolà L. (2010). Neuropharmacology of the essential oil of bergamot. *Fitoterapia*.

[4] Buchbauer G., Jirovetz L., Jager W., Plank C., Dietrich H. (1993). Fragrance compounds and essential oils with sedative effects upon inhalation. *Journal of Pharmaceutical Sciences*.

[5] Jimbo D., Kimura Y., Taniguchi M., Inoue M., Urakami K. (2009). Effect of aromatherapy on patients with Alzheimer's disease. *Psychogeriatrics*.

[6] Sadakane Y., Konoha K., Kawahara M. (2005). Protective activity of mango (*Mangifera indica* L.) fruit against a zinc-induced neuronal cell death is independent of its antioxidant activity. *Trace Nutrients Research*.

[7] Mellon P. L., Windle J. J., Goldsmith P. C., Padula C. A., Roberts J. L., Weiner R. I. (1990). Immortalization of hypothalamic GnRH neurons by genetically targeted tumorigenesis. *Neuron*.

[8] Mizuno D., Kawahara M., Lesieur C. (2014). Oligomerization of proteins and neurodegenerative diseases. *Oligomerization of Chemical and Biological Compounds*.

[9] Mizuno D., Kawahara M. (2013). The involvement of endoplasmic reticulum-stress in zinc neurotoxicity and the pathogenesis of vascular type senile dementia. *International Journal of Molecular Sciences*.

[10] Kanayama Y., Enomoto S., Irie T., Amano R. (2005). Axonal transport of rubidium and thallium in the olfactory nerve of mice. *Nuclear Medicine and Biology*.

[11] Kawahara M., Kuroda Y., Arispe N., Rojas E. (2000). Alzheimer's *β*-amyloid, human islet amylin, and priori protein fragment evoke intracellular free calcium elevations by a common mechanism in a hypothalamic GnRH neuronal cell line. *The Journal of Biological Chemistry*.

[12] Kawahara M., Sadakane Y., Koyama H., Konoha K., Ohkawara S. (2013). D-histidine and L-histidine attenuate zinc-induced neuronal death in GT1-7 cells. *Metallomics*.

[13] Campbell A. (2002). The Potential role of aluminum in Alzheimer's disease. *Nephrology Dialysis Transplantation*.

[14] Tiwari M., Kakkar P. (2009). Plant derived antioxidants—geraniol and camphene protect rat alveolar macrophages against t-BHP induced oxidative stress. *Toxicology in Vitro*.

[15] Kawahara M., Kato M., Kuroda Y. (2001). Effects of aluminum on the neurotoxicity of primary cultured neurons and on the aggregation of beta-amyloid protein. *Brain Research Bulletin*.

[16] Mizuno D., Konoha-Mizuno K., Mori M. (2015). Protective activity of carnosine and anserine against zinc-induced neurotoxicity: a possible treatment for vascular dementia. *Metallomics*.

[17] Hashimoto M., Inoue S., Muramatsu M., Masliah E. (1997). Estrogens stimulate tamoxifen-induced neuronal cell apoptosis *in vitro*: a possible nongenomic action. *Biochemical and Biophysical Research Communications*.

[18] Nakamura A., Nagata T., Kawahara M. (2007). Effects of essential oils in the proliferation and degeneration of immortalized hypothalamic neurons. *The Japanese Journal of Taste and Smell Research*.

[19] Howes M.-J. R., Houghton P. J., Barlow D. J., Pocock V. J., Milligan S. R. (2002). Assessment of estrogenic activity in some common essential oil constituents. *Journal of Pharmacy and Pharmacology*.

[20] Roberto D., Micucci P., Sebastian T., Graciela F., Anesini C. (2010). Antioxidant activity of limonene on normal murine lymphocytes: relation to H_2_O_2_ modulation and cell proliferation. *Basic and Clinical Pharmacology and Toxicology*.

[21] Murali R., Karthikeyan A., Saravanan R. (2013). Protective effects of D-limonene on lipid peroxidation and antioxidant enzymes in streptozotocin-induced diabetic rats. *Basic & Clinical Pharmacology & Toxicology*.

[22] Shinomiya M., Kawamura K., Tanida E. (2012). Neurite outgrowth of PC12 mutant cells induced by orange oil and d-limonene via the p38 MAPK pathway. *Acta Medica Okayama*.

[23] Zhuang S.-R., Chen S.-L., Tsai J.-H. (2009). Effect of citronellol and the Chinese medical herb complex on cellular immunity of cancer patients receiving chemotherapy/radiotherapy. *Phytotherapy Research*.

